# Technical feasibility of automated blur detection in digital mammography using convolutional neural network

**DOI:** 10.1186/s41747-024-00527-0

**Published:** 2024-11-18

**Authors:** S. Nowakowska, V. Vescoli, T. Schnitzler, C. Ruppert, K. Borkowski, A. Boss, C. Rossi, B. Wein, A. Ciritsis

**Affiliations:** 1 Department of Diagnostic and Interventional Radiology, University Hospital Zürich, Zürich, Switzerland; 2 b-rayZ AG, Schlieren, Switzerland; 3 Kantonsspital Aarau, Aarau, Switzerland; 4 Screening Aachen–Düren–Heinsberg, Aachen, Germany; 5 Present Address: GZO AG Spital Wetzikon, Wetzikon, Switzerland

**Keywords:** Artificial intelligence, Deep learning, Digital mammography, Image processing (computer-assisted), Quality assurance

## Abstract

**Background:**

The presence of a blurred area, depending on its localization, in a mammogram can limit diagnostic accuracy. The goal of this study was to develop a model for automatic detection of blur in diagnostically relevant locations in digital mammography.

**Methods:**

A retrospective dataset consisting of 152 examinations acquired with mammography machines from three different vendors was utilized. The blurred areas were contoured by expert breast radiologists. Normalized Wiener spectra (nWS) were extracted in a sliding window manner from each mammogram. These spectra served as input for a convolutional neural network (CNN) generating the probability of the spectra originating from a blurred region. The resulting blur probability mask, upon thresholding, facilitated the classification of a mammogram as either blurred or sharp. Ground truth for the test set was defined by the consensus of two radiologists.

**Results:**

A significant correlation between the view (*p* < 0.001), as well as between the laterality and the presence of blur (*p* = 0.004) was identified. The developed model AUROC of 0.808 (95% confidence interval 0.794–0.821) aligned with the consensus in 78% (67–83%) of mammograms classified as blurred. For mammograms classified by consensus as sharp, the model achieved agreement in 75% (67–83%) of them.

**Conclusion:**

A model for blur detection was developed and assessed. The results indicate that a robust approach to blur detection, based on feature extraction in frequency space, tailored to radiologist expertise regarding clinical relevance, could eliminate the subjectivity associated with the visual assessment.

**Relevance statement:**

This blur detection model, if implemented in clinical practice, could provide instantaneous feedback to technicians, allowing for prompt mammogram retakes and ensuring that only high-quality mammograms are sent for screening and diagnostic tasks.

**Key Points:**

- Blurring in mammography limits radiologist interpretation and diagnostic accuracy.
- This objective blur detection tool ensures image quality, and reduces retakes and unnecessary exposures.
- Wiener spectrum analysis and CNN enabled automated blur detection in mammography.

**Graphical Abstract:**

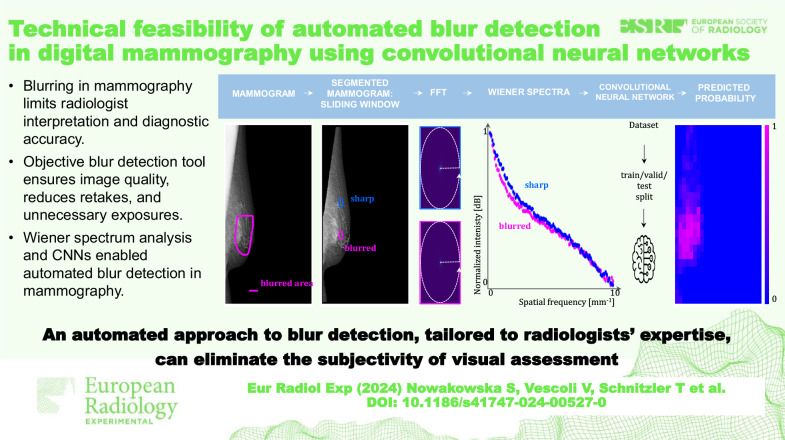

## Background

A reliable and automatic assessment of mammogram quality immediately after the acquisition allows for an immediate retake, reducing the recall rate, patient anxiety, radiologists’ workload, and costs. It also allows for building large databases of high-quality data thus enabling the development of reliable AI algorithms. One of the prevalent quality issues in mammography is blur [1], which can be caused by clamping paddle relaxation and patient motion [1–5] leading to a local or global blurring of a mammogram [1]. This artefact, depending on its location and breast tissue density, may limit diagnostic performance [3, 6]. Consequently, country-specific quality assurance systems, such as those in Austria, Germany, Norway, and the UK, include the absence of blur as one of the criteria, which an image must satisfy [7–11].

Currently, the identification of blur relies on visual assessment and is prone to high inter-reader variability. In a study conducted by Ma et al [6], in which simulated motion blur was applied to entire mammograms, the mean κ value was 0.26, indicating a fair inter-reader agreement [12]. Up to now, there is a very limited number of published studies concerning automatic and standardized motion blur detection in mammography. Kammona et al [13] trained machine learning models to detect regions with artificial blur, speculating that the simulation might have affected the quantum noise inherently present in the real-world data.

In another study, Hill et al [14] demonstrated that the normalized Wiener spectrum (nWS), also known as the normalized noise power spectrum, providing information about the intensity of spatial frequencies, holds the potential for blur detection. To calculate nWS for a given region of interest (ROI) of a mammogram, a windowing function, reducing the signal intensity at the ROI edges, is first applied. This is followed by the calculation of the normalized noise power spectrum, based on fast Fourier transform. The initial windowing step is performed to eliminate the ringing effect in the noise power spectrum. Subsequently, a radial average is calculated, deriving nWS. Hill et al [14], through the analysis of the extracted nWS, demonstrated that blurring causes a reduction in the intensity of spatial frequencies in the range of 1.6–2.4 mm^-^^1^. Importantly, in that work, 25 four-view mammography exams from a single vendor were utilized, and an iterative optimization procedure for multivariate linear regression against expert readers was performed. A comparative assessment within the patient study was performed, as it was hypothesized that nWS might exhibit significant variation based on tissue composition and complexity at a local scale within a given mammogram, as well as between breasts with different densities.

The primary objective of our study was to evaluate the technical feasibility of implementing an automated blur detection system in digital mammography using deep learning and real-world mammography data acquired with machines produced by various vendors and annotated by expert radiologists. In pursuit of this objective, we focused on creating an effective nWS postprocessing method coupled with convolutional neural network (CNN) model development, both enabling comparisons across diverse examinations. Importantly, deep learning models are well-suited for this task due to their capacity to learn complex patterns and nuances from highly diverse medical datasets, enabling them to mimic expert radiologists’ decision-making processes [15, 16].

## Methods

### Patient data

The data were retrieved from the local Picture Archiving and Communication System. All data were completely anonymized, and it is not possible to retrieve any personal information about the individual women. All participants examined have seen and signed an information letter in which they declare that the results of the examination may be used for scientific or educational purposes in accordance with the requirements of the General Data Protection Regulation.

The inclusion criteria were as follows: (1) age above 50 years, (2) absence of implants, and (3) presence of a blurred area within the breast in at least one mammogram from the study. Mammograms featuring blurred areas within the pectoralis muscle, folds, skin, and nipple were excluded. For mammograms with blurring within the breast interior, inclusion criteria focused on whether any findings might be obscured or distorted [6]. The examination selection was performed by BW, a board-certified radiologist with over 35 years of experience in breast imaging, based on the running screening reporting by experienced screening radiologists. The final dataset consisted of 152 mammography examinations (764 mammograms including the retakes) conducted between June 2019 and February 2023. The dataset included normal mammograms, as well as mammograms with benign and/or malignant findings. The patients’ age was 60 ± 6 years (mean ± standard deviation).

The data were acquired with different mammography machines: Siemens Revelation, and Fuji Amulet, as well as with Sectra MDM (models 1, 3, and 4). Most of the mammograms, *i.e*., 688/764 (90%), were acquired with a resolution of 4,915 × 5,355 pixels and 508 pixels/inch (*i.e*., 0.05 mm/pixel; see Supplementary Table S1 for details).

### Blur labelling and correlation with view, laterality, and compression force

For blur labelling, mammograms were presented to readers as images in Digital Imaging and Communications in Medicine−DICOM format. Annotations were conducted on 5-megapixel displays (RX560-MD RadiForce, EIZO Co, Ishikawa, Japan), adhering to reading conditions compliant with the DIN 6868-157 standard [17]. Standard magnifications were employed, with the additional capability to zoom to full resolution. BW (Reader 1) board-certified radiologist with over 35 years of experience in breast imaging, labelled the whole dataset by contouring blurred areas through polygons. The median polygon area amounted to 26.5 cm^2^ (interquartile range 18.2–44.6 cm^2^). Furthermore, TS (Reader 2), a senior radiology resident with over 7 years of experience in breast imaging, labelled the data in a test set (159 mammograms from 31 patients) with contours. A software using postscript markup developed by B.W. was used for the data annotation.

We assessed the correlation between the presence of blur and mammography view and laterality as specified below in the Statistics section.

### Image preprocessing

**Fig. 1:**
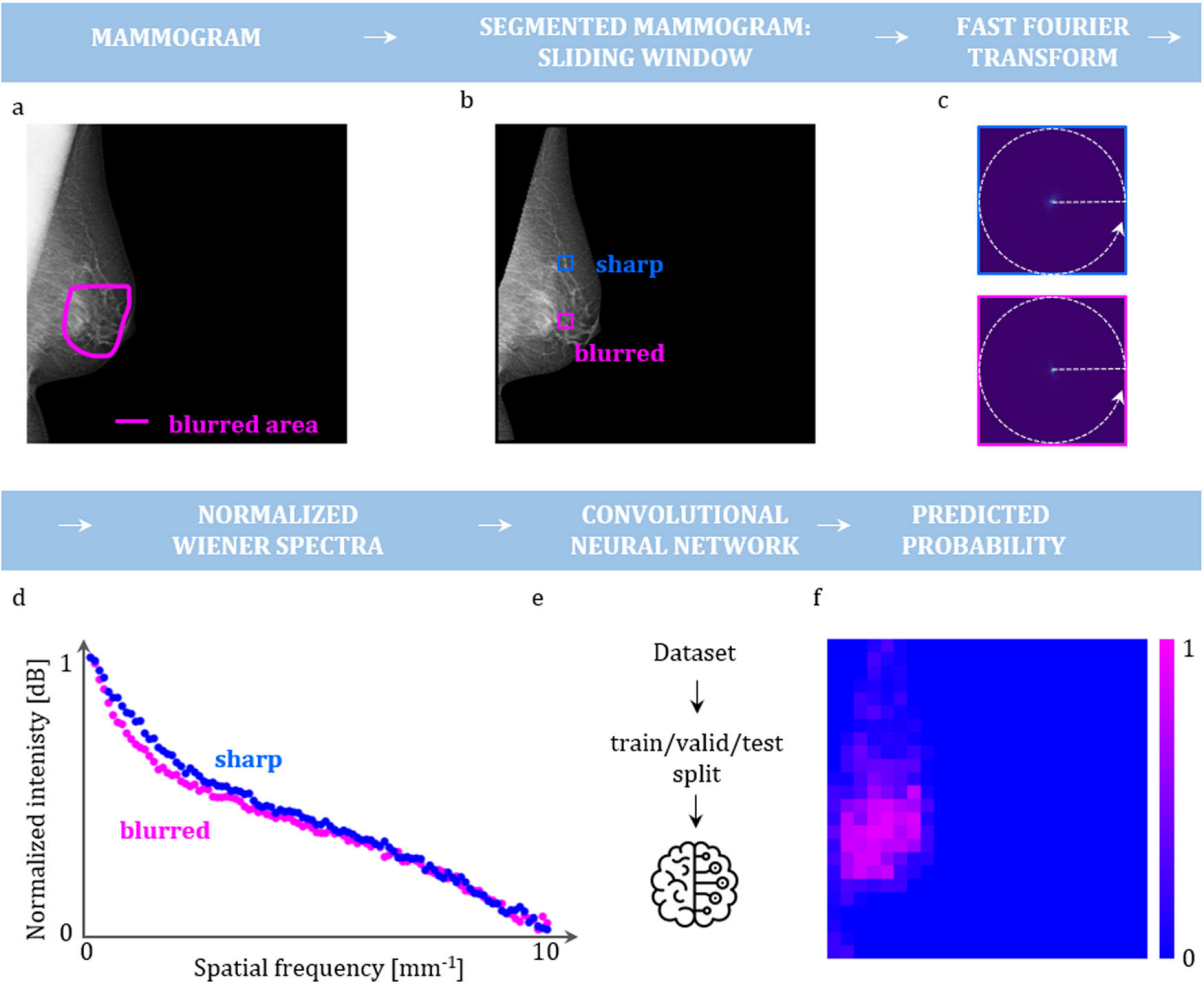
Schematic representation of the model development pipeline. **a** Mammograms with a delineated blurred area by expert radiologists serve as input. **b** The pectoralis muscle and the skin are removed. Subsequently, a window is slid over each mammogram, and at each location, a fast Fourier transform is performed (**c**). **d** A radial average is calculated serving as a basis for nWS computation. **e** The extracted nWS from the whole dataset is used for training, validation and testing of the CNN. **f** A probability map for each mammogram from a test set is obtained

A schematic illustration of the model development process is presented in Fig. 1. The labelled mammograms served as an input. In the first step all mammograms were rescaled to 4,915 × 4,915 pixels by adding pixels with intensity 0: for images with an “L” laterality, pixels were added to the right, while for those with “R” laterality, pixels were added to the left. Subsequently, intensity normalization to the 0–1 range was done. Next, the pectoralis muscle was removed with a b-rayZ AG proprietary deep-learning model, followed by the removal of background and skin using thresholding and contour-finding computer vision algorithms from OpenCV 4.9 Python library (*i.e.*, cv2.threshold, cv2.findCountours).

### Wiener spectra extraction

First, to assess the validity of nWS as a blur indicator, squared ROIs (1,000 × 1,000 pixels) were manually placed over both sharp and blurred regions, and the nWS spectra were extracted and compared. In the second phase, a fixed-size window (200 × 200 pixels) was slid across each image in the dataset, and nWS spectra were extracted from each window (see Fig. 1). For the details about the spectra extraction see Supplementary Section S1. The spectra extracted from all the mammograms resulted in an nWS dataset containing 4,379 spectra for a blurred region and 125,799 spectra for a sharp region.

### Dataset splitting and model training

Following best practices in model development [18], the dataset was split into two parts: the first part containing ~80% (103,948 of 130,178) of the data was used for hyperparameter tuning using 5-fold cross-validation, whereas the second part was retained to be used as a “hold-out test set” after the cross-validation was accomplished. The best hyperparameters were used to train the final model with the first part being used for training and validation, whereas the second part for testing. A schematic illustration of all the dataset splits is provided in Supplementary Fig. S1. All the dataset splits, including cross-validation, were performed in a patient-stratified way. The distribution of the classes in the splits during the development of the final model is illustrated in Supplementary Fig. S2. During each training, to compensate for the class imbalance weighted cross entropy loss was used. Further details regarding the training are described in Supplementary Section S2.

### Statistical analysis and model evaluation

We assessed the correlation between the presence of blur and mammography view and laterality using the χ^2^ test of independence, the correlation between the presence of blur and the compression force using the Spearman correlation coefficient (ρ), as the compression force did not follow the normal distribution (Shapiro–Wilk statistics 0.97, *p* < 0.0001).

The trained CNN model outputs a probability of a given nWS spectrum originating from a blurred region. For a given hyperparameter set, 5-fold cross-validation was conducted resulting in five distinct models. For each model, the area under the receiver operating characteristic curve (AUROC) for the corresponding test set was computed. To calculate the AUROC statistics, bootstrap resampling with replacement (*n* = 10,000) was used, generating AUROC values for each resample and determining 95% confidence intervals (CIs). A *p*-value was computed using Student’s *t*-test (AUROC > 0.5) on the bootstrapped values. This process yielded an average AUROC score for each hyperparameter set. The hyperparameter set with the highest AUROC was selected to train the final model. The hyperparameter set achieving the best performance consisted of a single layer CNN with 128 filters having kernel size equal to 3 and stride equal to 1, followed by batch normalization layer and a dropout layer with rate of 0.3, utilizing the Adam optimizer with a learning rate of 0.00001, and employing a batch size of 50.

After a final model was trained, confusion matrices were obtained for various probability thresholds with 95% CIs with *p*-values calculated similarly to the previous case. The threshold resulting in the highest true positive and true negative percentage was chosen. Ultimately, the chosen threshold for the window’s probability amounted to 0.45.

Subsequently, the chosen threshold was applied to the blur probability map generated for each mammogram within the test set. Windows with lower probabilities were then discarded. Additionally, a minimum requirement of ten interconnected windows was established to prevent the classification of tiny regions as blurred, considering that such regions lack diagnostic relevance. This criterion ensured that the blurred regions resembled the polygon labels provided by the radiologists. Furthermore, a minimum average probability criterion for the remaining windows was set. This criterion, amounting to 0.65, provided an additional layer of scrutiny, mirroring the considerations made in determining whether a retake of the mammogram is deemed necessary. Subsequently, mammograms that met the established criteria were considered blurred.

In the last phase, confusion matrices at the mammogram level were generated. The computation of 95% CIs and *p*-values followed the same approach as that employed for window-level confusion matrices. Furthermore, the weighted accuracy was determined, by adjusting class weights inversely proportional to their frequencies for a balanced dataset to exemplify the task of blur detection. The null hypothesis for the *t*-test was set to > 0.5. For Cohen’s κ, the null hypothesis was set to κ = 0.

### Model explainability

To gain further insight into the predictions of the CNN model and assess whether certain frequency ranges significantly influence these predictions, an explainability analysis using Shapley values [19, 20] was performed. Shapley values are a concept from cooperative game theory that allocates a fair distribution of payoffs to players based on their contribution to the total payout. In the context of machine learning, Shapley values are used to explain the contribution of each feature to the prediction of a model [21]. The details of the analysis are provided in Supplementary Section S3.

The statistical analysis and model explainability was performed by the first author. Values of *p* lower than 0.050 were considered as significant. The numbers in parentheses across the manuscript refer to 95% CIs with *p*-value < 0.001.

### Model inference time

The inference time of the model was assessed on a notebook equipped with 16 GB of RAM. Predictions for a mammogram of size 4,915 × 4,915 pixels were performed ten times, and the mean and standard deviation of the inference times were subsequently calculated.

### Language optimization

The manuscript text was stylistically optimized using large language models, specifically Chat GPT (OpenAI) and Gemini (Google).

## Results

### Correlation between blur and mammography view, laterality, and compression force

**Fig. 2:**
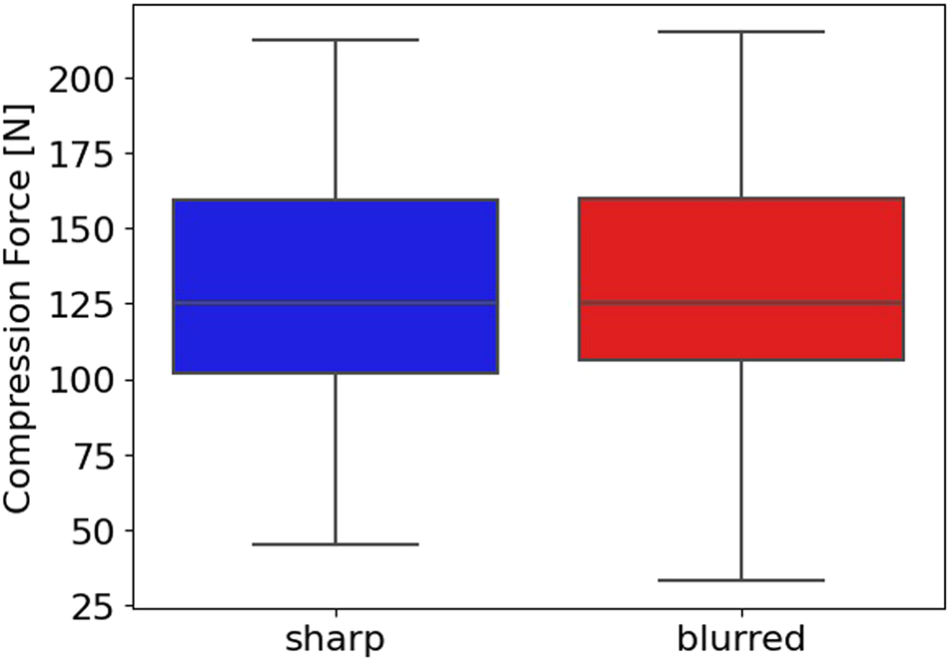
Correlation between blurring and the compression force: a boxplot summarizing the distribution of the compression force used for the acquisition of mammograms labelled as sharp (blue) and blurred (red)

The contingency tables for the presence of blurring and the view/laterality (Table 1) served as an input for the χ^2^ test of independence. The results indicate a statistically significant correlation between the view and the presence of a blurring (*p*-value < 0.001), as well as between the laterality and the presence of blur (*p*-value = 0.004). Specifically, the left mediolateral oblique view (MLO) view predominantly featured blurring. The Spearman correlation coefficient calculated for blurring and compression force revealed no significant correlation: ρ = -0.03, *p*-value = 0.338 (see Fig. 2).

**Table 1:** Contingency table for view and laterality

	View		Laterality	
Class	Craniocaudal	Mediolateral oblique	Right	Left
Blurred	24	134	60	98
Sharp	306	300	310	296

### Comparison of nWS spectra extracted from blurred and sharp areas

**Fig. 3:**
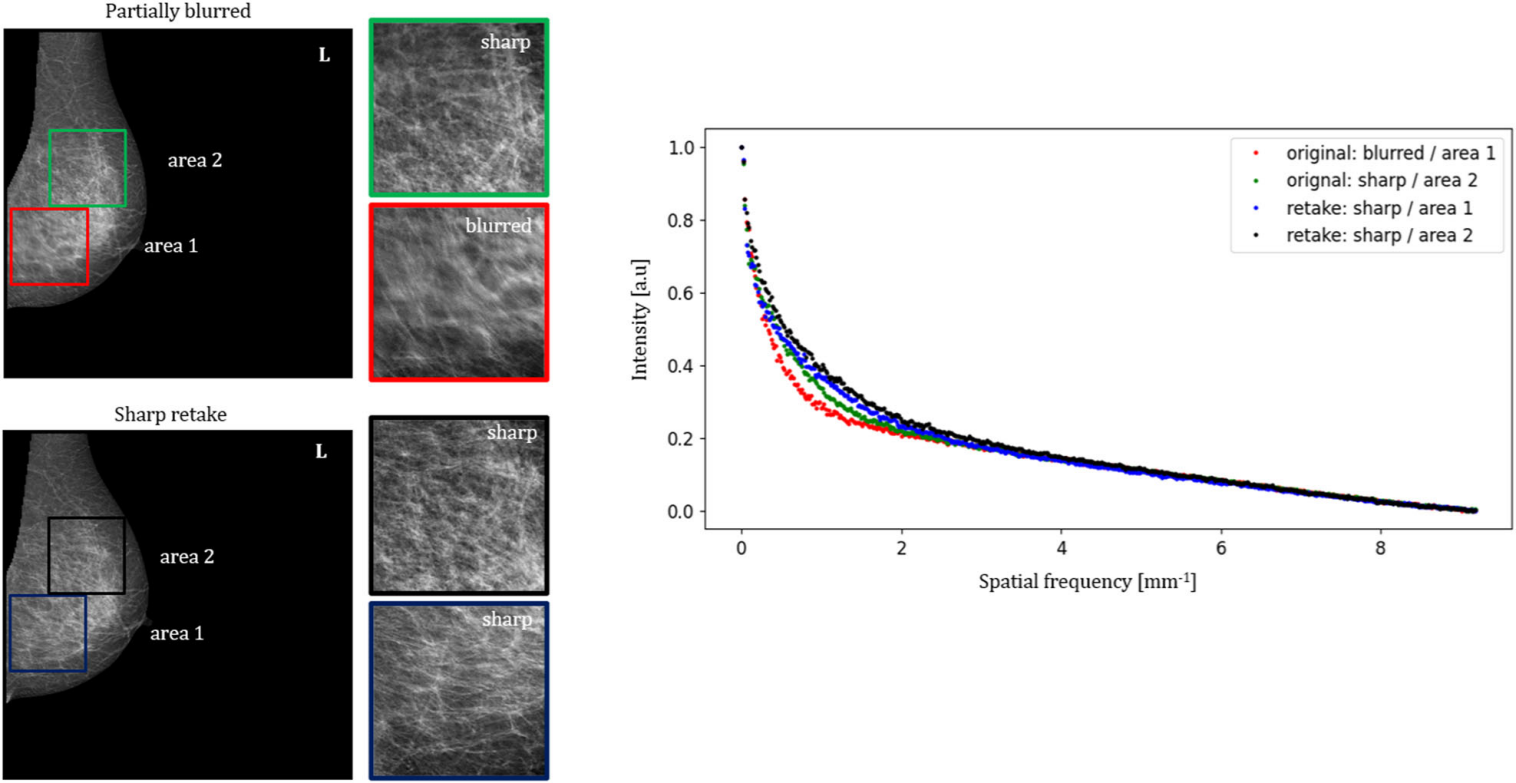
Comparison of nWS extracted from blurred to sharp areas. A partially blurred left mediolateral oblique mammogram and a corresponding sharp retake with the chosen squared regions of interest, from which the nWS spectra were extracted

In Fig. 3, a comparison of nWS extracted from blurred and sharp areas is presented. A left MLO mammogram was labelled as partially blurred and a corresponding sharp retake was included. Manually placed squared ROIs cover two areas: Area 1, including the blurred region in the original mammogram and its corresponding sharp region in the retake, and Area 2, including sharp regions in both the original and the retaken mammograms. The nWS spectrum originating from the blurred ROI has the lowest intensity in the lower spatial frequencies.

### CNN model evaluation

**Fig. 4:**
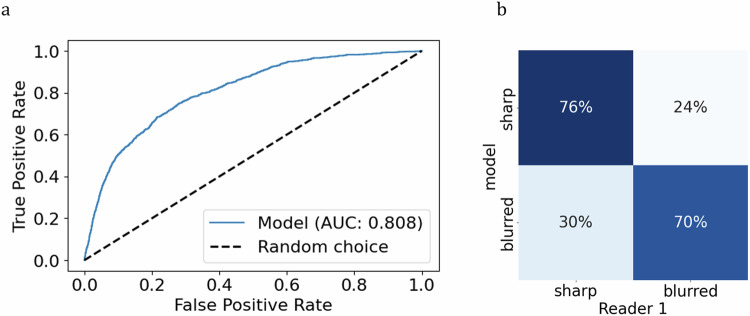
Model’s evaluation on the window level. **a** Receiver operating characteristics analysis with the reported AUROC. **b** Confusion matrix at the chosen threshold of 0.45

The CNN model trained on the entire dataset with optimized hyperparameters, previously determined through cross-validation, achieved AUROC of 0.808 (0.794–0.821) (Fig. 4a). Upon applying a threshold of 0.45 to the model’s probability output, 76% of nWS were correctly classified as originating from sharp regions (75–76%) and 70% as originating from blurred regions (67–73%) (Fig. 4b).

**Fig. 5:**
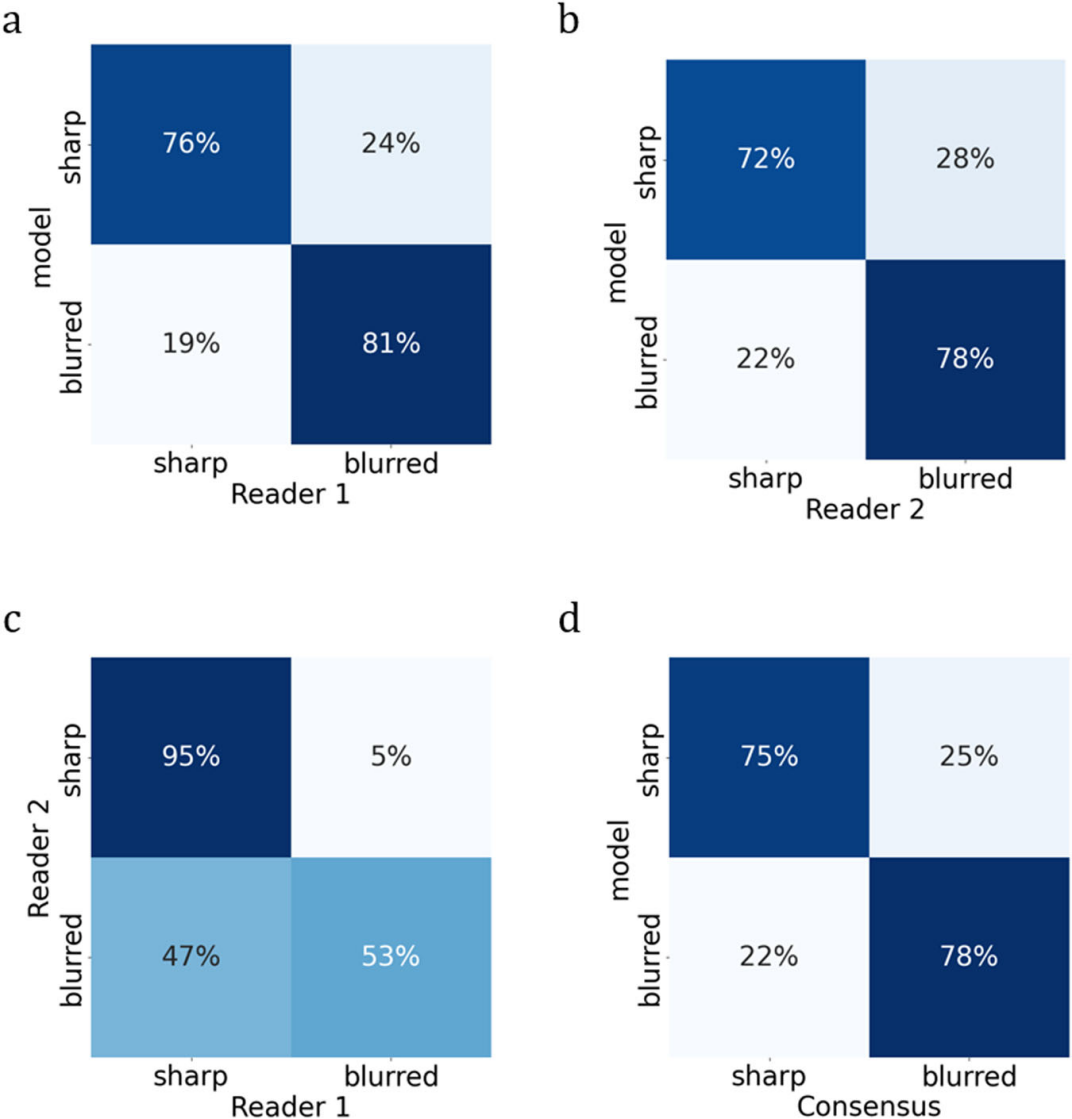
Readers’ and model’s evaluation at the mammogram level: **a** Reader 1 *versus* model, (**b**) Reader 2 *versus* model, (**c**) Reader 1 *versus* Reader 2, and (**d**) Consensus *versus* model

At the image level (see Fig. 5 and Table 2), the model exhibited alignment with Reader 1 in 81% (67–94%) and with Reader 2 in 78% (60–94%) of mammograms classified as blurred, while the readers achieved consensus in 53% (35–70%) of such cases. In sharp mammograms, the model achieved agreement in 76% (68–84%) with Reader 1 and 72% (64–80%) with Reader 2, whereas readers reached a consensus in 95% (91–98%) of cases. The model’s weighted accuracy computed with reference to Reader 1 amounted to 0.840 (0.693–0.966) and with reference to Reader 2 to 0.808 (0.623–0.737). The Cohen’s κ for both readers amounted to 0.45 (0.30–0.59) indicating fair/moderate agreement. The wide 95% CI indicates slight/fair/moderate agreement of the model with the readers (see Table 2).

**Table 2:** Readers’ and model’s evaluation at the mammogram level

	True positives	True negatives	Weighted accuracy^a^	Cohen κ
Model *versus* Reader 1	26/32 81% (67–94%)	97/127 76% (68–84%)	0.027 + 0.813 0.840 (0.693–0.966)	0.45 (0.30–0.59)
Model *versus* Reader 2	18/23 78% (60–94%)	98/136 72% (64–80%)	0.025 + 0.783 0.808 (0.623–0.972)	0.31 (0.17–0.46)
Reader 2 *versus* Reader 1	17/32 53% (35–70%)	121/127 95% (91–98%)	0.033 + 0.530 0.563 (0.387–0.737)	0.54 (0.35–0.70)
Model *versus* consensus	26/32 78% (67–83%)	97/127 75% (67–83%)	0.25 + 0.579 0.829 (0.680–1.000)	0.32 (0.17–0.49)

^a^ Class weights are assigned inversely proportional to their respective frequencies as assigned by Reader 1. The 95% CI values are indicated in parentheses. All *p*-values are below 0.001

In consideration of the subjectivity of blur detection among the readers, the model also underwent evaluation in instances where both readers reached a consensus. The model aligned with the consensus that 78% (67–83%) of mammograms were classified as blurred and 75% (67–83%) identified as sharp. The weighted accuracy amounted to 0.829 (0.680–1.000), whereas the Cohen’s κ to 0.32 (0.17–0.49).

**Fig. 6:**
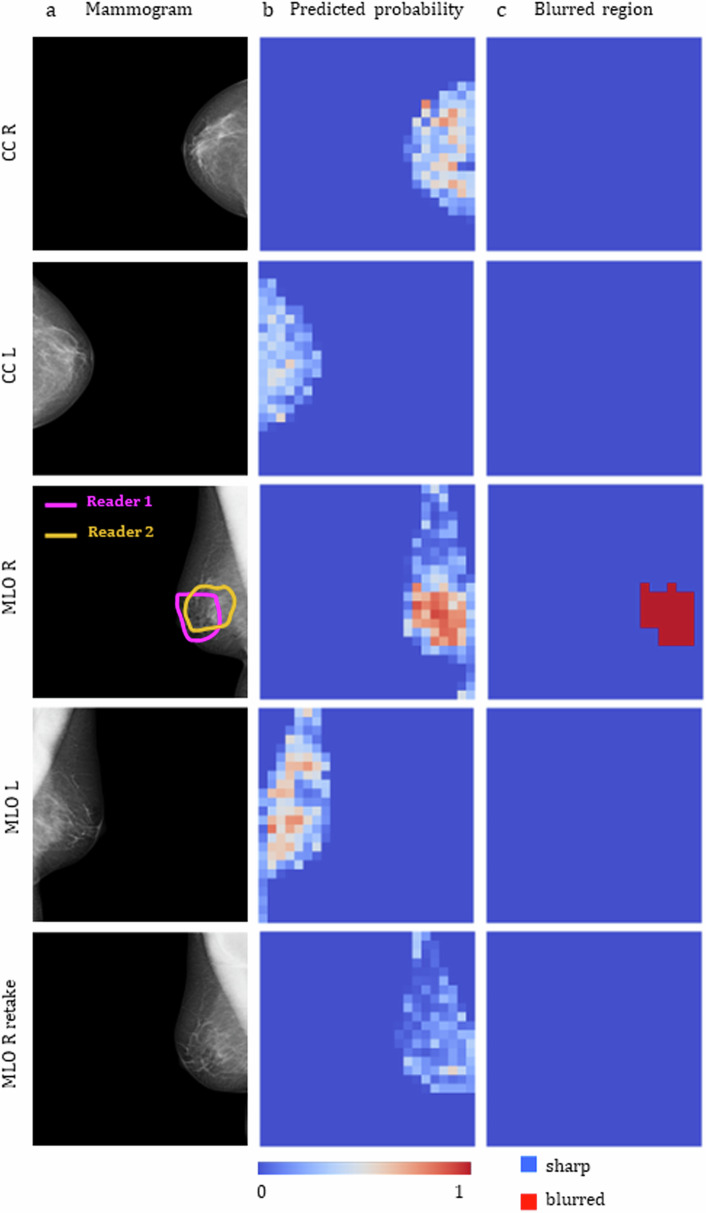
Blur detection results for a mammographic examination contained in the test set. **a** The right MLO mammogram in the original examination was labelled by both Readers 1 and 2 as blurred, the retake of this view is also shown in the bottom line. **b** Blur probability maps output by the trained model. **c** Final blurred area classification obtained after thresholding. CC, Craniocaudal view; L, Left breast; MLO, Mediolateral oblique view; R, Right breast

In Fig. 6, blur detection results are shown for an examination from the test set. In this specific instance, the MLO view for the right breast exhibits partial blurring in a diagnostically relevant area, as indicated by the contours drawn by the readers. A retake of this view and laterality is also included. The predicted probabilities by the CNN model on the window level for each mammogram are shown in the second column. The final blurred area, obtained after thresholding, is shown in the third column. The right MLO mammogram was correctly identified as blurred, with the blurred area closely resembling the contours outlined by the radiologists. The remaining mammograms were correctly classified as sharp.

### Model explainability

**Fig. 7:**
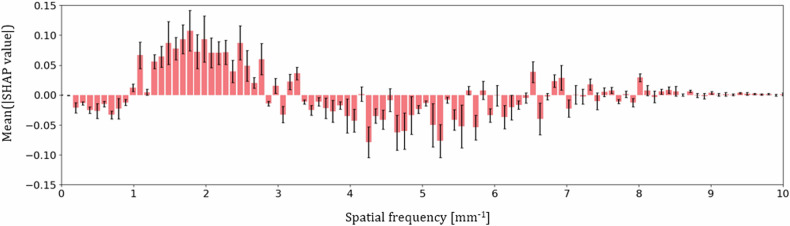
Feature importance for model predictions. Average Shapley values calculated for each spatial frequency bin with corresponding standard errors depicted by black arrows

The plot displayed in Fig. 7 summarizes the analysis of the Shapley values for windows contained in a test set and classified by the model as blurred, *i.e*., having the probability above the chosen threshold value of 0.45. Spatial frequency values with a mean Shapley additive explanations value above zero contribute to an increased probability of blur output by the model, while those below zero contribute to a decreased probability. Three frequency ranges can be identified in the plot: ~1–3 mm^-1^ increasing the probability of nWS spectra being classified as blurred ~0–1 mm^-1^ and ~3–6.5 mm^-1^ decreasing the probability.

### Model inference time

The model inference time amounted to 5.5 ± 0.6 s.

## Discussion

This study demonstrates the technical feasibility of implementing an automated blur detection system in digital mammography by combining Wiener Spectra extraction with CNN. A crucial aspect introduced in this work was the postprocessing step of the nWS model spectra involving normalization, which introduced an absolute scale, allowing for training the CNN model with spectra originating from different examinations, breast densities, and vendors, followed by the development of thresholding steps with uniform values for all the data, thereby removing the limitation of comparative assessment of the Wiener Spectra within a single examination as shown by Hill [14]. Owing to that, a broad applicability, essential for future integration into clinical practice was achieved.

An equally important consideration for clinical utility is the inference time, which in this study amounted to 5.5 ± 0.6 s. Such an inference time would allow for an immediate retake with a patient still present in an examination room.

The analysis revealed a significant correlation between the mammogram view and the presence of blur (*p* < 0.001), with the MLO view exhibiting a higher prevalence of blurring artefacts. This phenomenon can be attributed to the acquisition process of the MLO view, which requires a patient to maintain a forced posture for around 30 s. This uncomfortable position can lead to micro-movements, which can cause blurring. This is not the case in the craniocaudal view.

It is important to note that the compression force is typically adjusted depending on the patient-specific factors such as pain threshold, breast elasticity, and size [22], which can influence blurring and thus also the case selection.

Various factors reported in the literature influence blur detection, including blur area size, reader experience, and acuity [3], as well as screen resolution [11]. In our study, the agreement between the readers was fair/moderate (κ = 0.45, 0.30–0.59). In another study conducted with real-world data, near-perfect inter-reader agreement on the blur presence (κ = 0.84) was reported, while the agreement for retake necessity was fair (κ = 0.22) [14]. Noteworthy, the model developed in our study, struck a balance between the two readers. When compared with both Reader 1 and Reader 2 the model exhibited higher sensitivity at the expense of lower specificity (see Fig. 5a, b*versus* c). The model exhibited similar performance when assessed on instances where a consensus was reached.

The manually placed squared ROIs indicate that blurring manifests as a decrease in intensity within the low spatial frequencies (see Fig. 3). The analysis of the Shapley values revealed that the spatial frequency range contributing to an increased blur probability output by the model is ~1–3 mm^-1^, consistent with the findings from manually placed ROIs. This range also aligns with the results obtained by Hill et al [14], who identified a spatial frequency range of 1.6–2.4 mm^-1^ as the most informative for blur detection. Additionally, considering the scarcity of data and the time-intensive nature of searching, retrieving, and annotating the data, the insights into the physics of blur provided in this study can be utilized to develop strategies for the introduction of realistic artificial blur to sharp mammograms, paving the way for various deep learning models’ development.

The main limitation of our study is the data are highly skewed towards sharp images and relatively small sample size, lack of external validation, and the fact that 80% of the dataset, used for cross-validation was labelled by a single, albeit highly experienced, radiologist. Furthermore, the current work did not assess the real clinical value of the model. Specifically, our study only references whether the images appear blurred or not, without evaluating how the detected blur impacts diagnostic quality or patient recall rates. In subsequent studies, these limitations will be overcome by significantly increasing the number of mammography images, featuring a broad range of resolutions and originating from various clinical sites, as well as by ensuring that annotation is performed by multiple expert radiologists. A deep learning model, developed on the larger dataset, will be evaluated clinically in a prospective study, both in diagnostics and screening settings. After successful clinical evaluation, in a diagnostic setting, when presented with a map indicating a blurred area in a mammogram (see Fig. 6c), a technician will be able to promptly consult the attending radiologist to determine if a retake is necessary. In this workflow scenario, the experienced radiologist will consider various factors including available views, breast density and the specific location of the blurred area, as well as the possibility of additional imaging. In this way, the risk of potential unwarranted x-ray exposure is minimized. In the screening setting, in which the radiographers are specifically trained to acquire high-quality mammograms and are solely responsible for retake decisions, the model can provide additional support in their decision-making process. In both settings, the need for a recall, which not only induces additional psychological stress for the patient but also increases costs for the facility, can be mitigated.

In conclusion, in this technical feasibility study, a blur detection deep learning model with broad applicability was developed and evaluated. Our findings suggest that a reliable method for detecting blur, utilizing feature extraction in frequency space, and customized to align with the clinical expertise of radiologists, could mitigate the subjectivity inherent in visual assessments. Future research will focus on evaluating the clinical impact on diagnostic accuracy and patient recall rates to fully establish the model’s value in diagnostic and screening settings.

## Supplementary information

**Additional file 1:** **Table S1** Resolution of the mammograms in the dataset. **Fig. S1 Dataset splitting:** The dataset was split into two parts: the first part containing ~ 80% of the data was used for hyperparameter tuning using 5-fold cross-validation (CV), whereas the second part was retained to be used as an “outer test set” after the CV was accomplished. **Fig. S2 Training of the final model:** The distribution of the classes in the training, validation and test set on the window level.

## Data Availability

The original data set retrieved from the Picture Archiving and Communication System, anonymized and analysed in the current study is not publicly accessible due to the confidentiality of patient data in accordance with the privacy policy of the Aachen–Düren–Heinsberg screening unit. Part of the dataset may be made available by the corresponding author to bona fide researchers for non-commercial purposes upon reasonable request.

## Abbreviations

AUROC: Area under the receiver operating characteristic curve
CI: Confidence interval
CNN: Convolutional neural network
MLO: Mediolateral oblique view
nWS: Normalized Wiener spectrum/spectra
ROI: Region of interest
