# Supplementary material for: Technical feasibility of automated blur detection in digital mammography using convolutional neural network
Source: Eur Radiol Exp. 2024 Nov 18;8:129. doi: 10.1186/s41747-024-00527-0 (PMC11574226; doi:10.1186/s41747-024-00527-0)
Supplement: Supplementary file 1 — Additional file 1: Table S1 Resolution of the mammograms in the dataset. Fig. S1 Dataset splitting: The dataset was split into two parts: the first part containing ~ 80% of the data was used for hyperparameter tuning using 5-fold cross-validation (CV), whereas the second part was retained to be used as an “outer test set” after the CV was accomplished. Fig. S2 Training of the final model: The distribution of the classes in the training, validation and test set on the window level. [file 41747_2024_527_MOESM1_ESM.pdf]

Technical feasibility of automated blur detection in digital mammography using convolutional neural network

ELECTRONIC SUPPLEMENTARY MATERIAL

Table S1 Resolution of the mammograms in the dataset

Resolution: columns x rows [px x px]	Number of mammograms
2364 x 3164	23
2682 x 3482	1
2800 x 3518	20
4728 x 5355	32
4915 x 5355	688

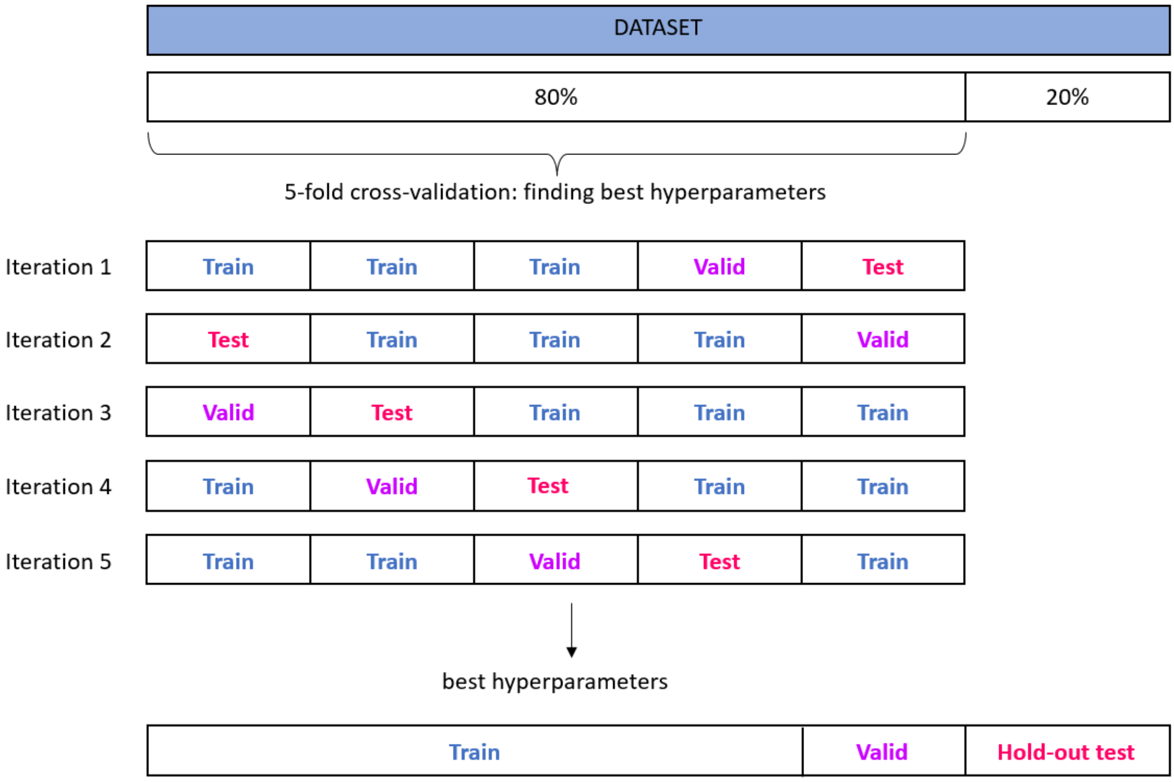

Fig. S1 Dataset splitting: The dataset was split into two parts: the first part containing ~ 80% of the data was used for hyperparameter tuning using 5-fold cross-validation (CV), whereas the second part was retained to be used as an "outer test set" after the CV was accomplished.

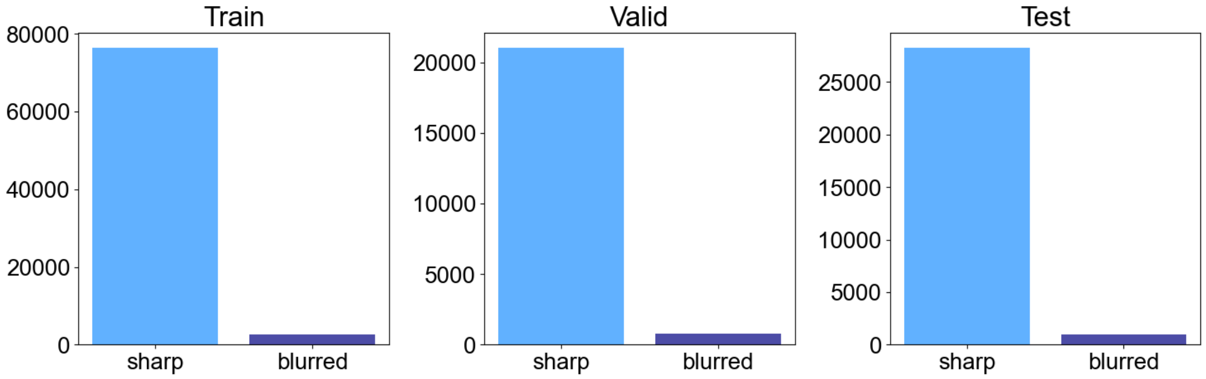

Fig. S2 Training of the final model: The distribution of the classes in the training, validation and test set on the window level.

Section S1: ***Wiener Spectra calculation***

Initially, a high-pass Butterworth filter with a cutoff frequency of 0.0001 was applied within the window. Next, the nWS was computed as in Ref. [1]. In the next step, the nWS spectra were post-processed by conversion to a dB scale and subsequent normalization. If at least 75% of the window area was within the contour delineated by Reader 1, it was considered to characterize a blurred region.

Section S2: ***Model training***

Prior to training, for comparability, the random seed was globally fixed, and the CNN model's weights were initialized utilizing glorot uniform method, whereas the biases with zero. A single training run was set to 150 epochs with early stopping ensuring that a model with the lowest validation loss was chosen for evaluation. The training was performed on a CPU with 16 GB RAM.

Section S3: ***Model explainability***

For this analysis, a Deep Explainer from SHAP Python library was used, as it is suitable for model architectures based on neural networks [2]. This explainer calculates Shapley values by integrating over background samples. These values represent contributions to the model output, ensuring they sum up to the difference between the expected model output on the background samples and the current model output [3]. Due to computational constrains, a randomly chosen subset of 2000 nWS spectra from the training set, half labeled as blurred and half as sharp, was used as a background. The explainer was then applied to a similarly chosen

subset of the test set to compute Shapley values. Their average was computed for each frequency bin together with a standard error.

Supplementary References

1. Hill ML, Whelehan P, Vinnicombe SJ, et al (2018) Development of an automated detection algorithm for patient motion blur in digital mammograms. In: Krupinski EA (ed) 14th International Workshop on Breast Imaging (IWBI 2018). SPIE, Atlanta, United States, p 50

2. Lundberg SM, Lee S-I (2017) A Unified Approach to Interpreting Model Predictions. In: Advances in Neural Information Processing Systems. Curran Associates, Inc.

3. shap.DeepExplainer — SHAP latest documentation. https://shap-lrjball.readthedocs.io/en/latest/generated/shap.DeepExplainer.html. Accessed 13 May 2024
